# Green Recycling of Waste Tire Rubber Crumb: Characterization and Water Treatment Applications

**DOI:** 10.3390/polym18202492

**Published:** 2026-10-12

**Authors:** Ahmed Hamad Alanazi, Tamer H. A. Hasanin, Saad Alrashdi, Amnah Salem Al Zbedy, Fahed A. Aloufi, Riyadh F. Halawani, Amr Mohammad Nassar

**Affiliations:** 1 Chemistry Department, College of Science, Jouf University, Sakaka 72341, Saudi Arabia; thhasanin@ju.edu.sa (T.H.A.H.); saalrashdi@ju.edu.sa (S.A.); 2 Department of Chemistry, Al-Qunfudah University College, Umm Al-Qura University, P.O. Box 1109, Al-Qunfudah 21912, Saudi Arabia; asazubidi@uqu.edu.sa; 3 Department of Environment, Faculty of Environmental Sciences, King Abdulaziz University, Jeddah 21589, Saudi Arabia; faloufi@kau.edu.sa (F.A.A.); rhalawani@kau.edu.sa (R.F.H.); 4 Water Research Center, King Abdulaziz University, P.O. Box 80200, Jeddah 21598, Saudi Arabia

**Keywords:** tire, sustainable, green, adsorption, water

## Abstract

Discarded tires are adding to the waste problem that exists worldwide and requires proper recycling methods. Rather than employing chemicals or energy-intensive thermal treatments, which leads to an increase in environmental pollution, in this work, the discarded tires are subjected to mechanical grinding into a fine powder form, resulting in recycled Waste Tire Rubber Crumb (WTRC), then used as an adsorbent for the removal of methylene blue (MB) dye from the polluted water. The use of the adsorbent is eco-friendly and cost-effective, which is beneficial for water treatment. The structural, thermal, and morphological characteristics of WTRC were studied with the use of X-ray diffraction (XRD), scanning electron microscopy with energy-dispersive X-ray spectroscopy (SEM/EDX), thermal gravimetric analysis (TGA), differential scanning calorimetry (DSC), transmission electron microscopy (TEM), dynamic light scattering (DLS), and zeta potential analysis. It was found that there was an elastic polymer matrix with inorganic additions of ZnO and CaCO_3_. Zeta potential measurements provided the value −32.5 mV at pH 7.0, demonstrating electrostatic repulsion of the particles and providing solid colloidal stability in aqueous media. In batch tests, WTRC proved to be a good adsorbent of MB, with up to 92.7% removal efficiency within 30 min. under the basic medium (pH 11.0, 1.2 g/50 mL dosage, and initial MB concentration of 5 mg/L ppm). The Langmuir model gave the best fit, with R^2^ = 0.982, which is more than Freundlich model, R^2^ = 0.957. The good fit of the Langmuir model indicates that adsorption predominantly occurred as a monolayer on relatively homogeneous adsorption sites on the surface of WTRC. The theoretical monolayer capacity (q_max_) is calculated as 0.95 mg/g. It is proved by the surface changes after the adsorption of MB, shown in FTIR and SEM pictures. In general, the raw WTRC can serve as a simple and economic adsorbent for dyes from industry.

## 1. Introduction

The increasing growth of the industrial sector has caused the emergence of synthetic dyes that enter into water systems [1]. The cationic dye methylene blue (MB) that is used widely in the textile, plastic, and printing industries constitutes a significant threat to the environment due to its high chemical stability, photostability, and toxicity [2]. The presence of colored organic dyes in water streams prevents the penetration of light into the water beds and affects the photosynthesis process, thus disturbing the ecosystem [3]. There is a need to find cost-effective methods for capturing dyes prior to their discharge into natural water sources.

Adsorption can be considered the easiest method of removing dyes due to the simplicity of this process, low-energy demand, and lack of secondary sludge produced during the treatment [4]. Nevertheless, conventional technologies based on the use of commercial adsorbents such as activated carbon turn out to be too expensive for mass production and regeneration [5]. Hence, it has become necessary to develop the use of various industrial and solid waste as adsorbents in accordance with the circular economy ideology [6].

One of the most problematic types of industrial solid waste is scrap automobile tires. Every year millions of tires become redundant, but their durability prevents them from being degraded easily. Scrap tires are commonly disposed of at landfills or illegal dumps, which causes environmental and health hazards [7]. Using scrap rubber as the material for water treatment is a solution to a difficult disposal problem. Converting used rubber into a usable substance for water treatment changes a continuous problem of disposal into an effective solution [8,9].

Unlike the prior method, which involves costly procedures such as pyrolysis and chemical activation for generating carbon char from the used tires [10,11,12,13,14], the process of using WTRC made through mechanical washing and grinding without burning waste tires is much cheaper and more environmentally friendly. Unlike other adsorbents that have a high degree of purity, WTRC is made up of natural rubber that has carbon black, cross-linked polymers and inorganic filler compounds, including zinc oxide and calcium carbonate [15,16].

The objective of this study was to investigate the feasibility of using WTCR as an untreated, low-cost, and sustainable adsorbent for the remediation of MB dye from contaminated water. To achieve this goal, WTCR was characterized using XRD, EDX, TGA/DSC, SEM, TEM, DLS, and zeta potential analyses to elucidate its physicochemical properties. In addition, the factors affecting the sorption process, such as adsorbent dosage, solution pH, initial MB concentration, and contact time, were examined to attain the optimal adsorption conditions. Equilibrium isotherm and kinetic studies were also investigated. The possible interactions between WTRC and MB dye were proposed.

## 2. Experimental

### 2.1. Materials

The car tire scraps were obtained from a car repair shop in Sakaka, Saudi Arabia. The car tire waste was shredded and then washed in distilled water while being heated to 70 °C for 30 min, then filtered and air-dried. MB dye was obtained from Sigma-Aldrich (St. Louis, MO, USA). The aqueous solutions were prepared using Milli-Q water.

### 2.2. Instruments

The SEM images were recorded by a Zeiss LEO Supra 55VP Field Emission SEM and a Zeiss 1530 (Carl Zeiss, Oberkochen, Germany) operated at 20 kV and a working distance of 10 mm. Powder X-ray diffraction spectra were recorded by the D8-Find Bruker diffractometer (Bruker, Billerica, MA, USA), which is equipped with a Cu Kα radiation source (λ = 1.5418 Å) operated at 40 kV and 40 mA. The FTIR measurements were performed with an IRTracer-100 SHIMADZU spectrophotometer (Shimadzu, Kyoto, Japan) in the range of 4000 cm^−1^ to 400 cm^−1^. Zeta potential analysis was done at room temperature by a NanoSight NS500 zeta sizer analyzer provided by Malvern Panalytical Ltd. (Panalytical Ltd., Malvern, Panalytical, Malvern, UK). The NanoSight NS500 Model Particle Size Analyzer used in DLS analysis is provided by Malvern Panalytical Ltd. (Malvern Panalytical, Malvern, UK). Absorption spectra were recorded by a Cary 60 UV-Vis spectrophotometer (Agilent Technologies, Santa Clara, CA, USA) in the range of 190–1100 nm. TEM images were captured by JOEL JEM-2100F (JEOL Ltd., Tokyo, Japan).

### 2.3. Adsorption Experiments

The efficiency of WTRC in wastewater treatment has been investigated using MB dye. The effects of several parameters (adsorbent masses, initial dye concentrations, contact time, and pH) on the adsorption process were investigated by adsorption experiments. In a 250 mL beaker, 50 mL of MB (1.0–50 mg/L) solution was combined with various adsorbent dosages in the range (0.1 to 1.5 g) and the mixture was agitated at room temperature in the range (5.0–180 min) within pH range (1.0–11) in the dark. 0.1 M of HCl and NaOH were used to adjust the pH. The mixture was agitated at 300 rpm throughout the adsorption experiments. The CTR was sieved to obtain particles within the size range of 0.25–0.50 mm (35–60 mesh).

The mixture was agitated and then centrifuged at 3000 rpm for 3 min. Next, a UV-Vis spectrophotometer with a wavelength of 665 nm was used to measure the dye concentration. Equation (1) was used to obtain the percentage of dye removal [17],

(1) $$R\;\%=\frac{C0-Ce}{C0}*100$$

where C_0_ is the initial MB concentration (mg/L) and Ce is the concentration of MB after treatment.

## 3. Results and Discussion

**Scheme 1:**
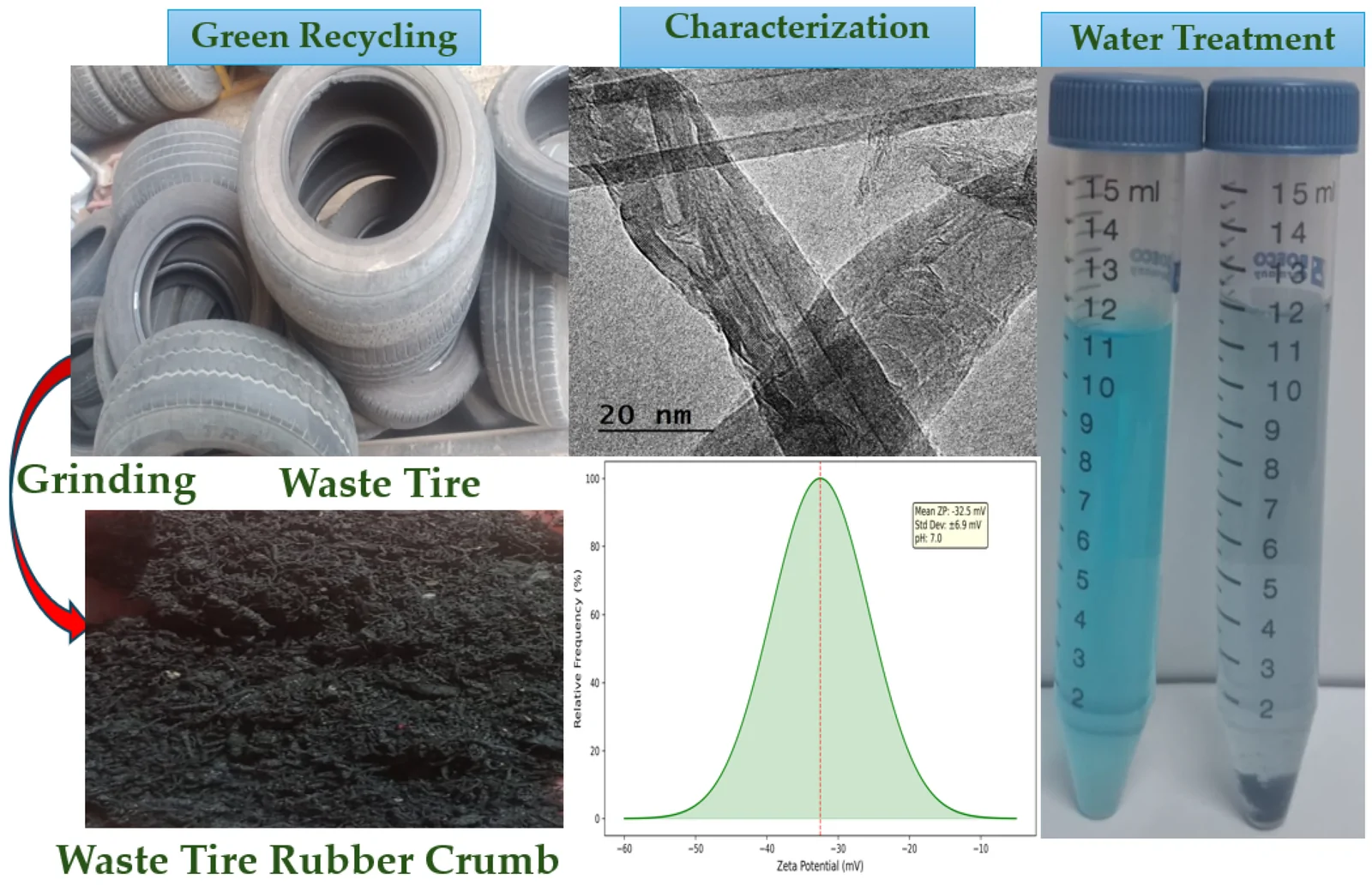
Green recycling of waste tires for wastewater treatment.

The current work describes a novel and ecofriendly process for wastewater purification using ground tire waste in its native form, without any required thermal treatment such as pyrolysis or incineration at high temperatures. This approach does not require any heating and hence reduces energy consumption and the generation of secondary emissions into the atmosphere. Furthermore, no acidic or alkaline treatment is required, minimizing chemical consumption and operational costs. It is worth mentioning that the obtained WTRC displays remarkable green process for the removal of MB dye from wastewater, Scheme 1.

### 3.1. Characterization of WTRC

#### 3.1.1. Crystallographic Structure (XRD)

**Figure 1:**
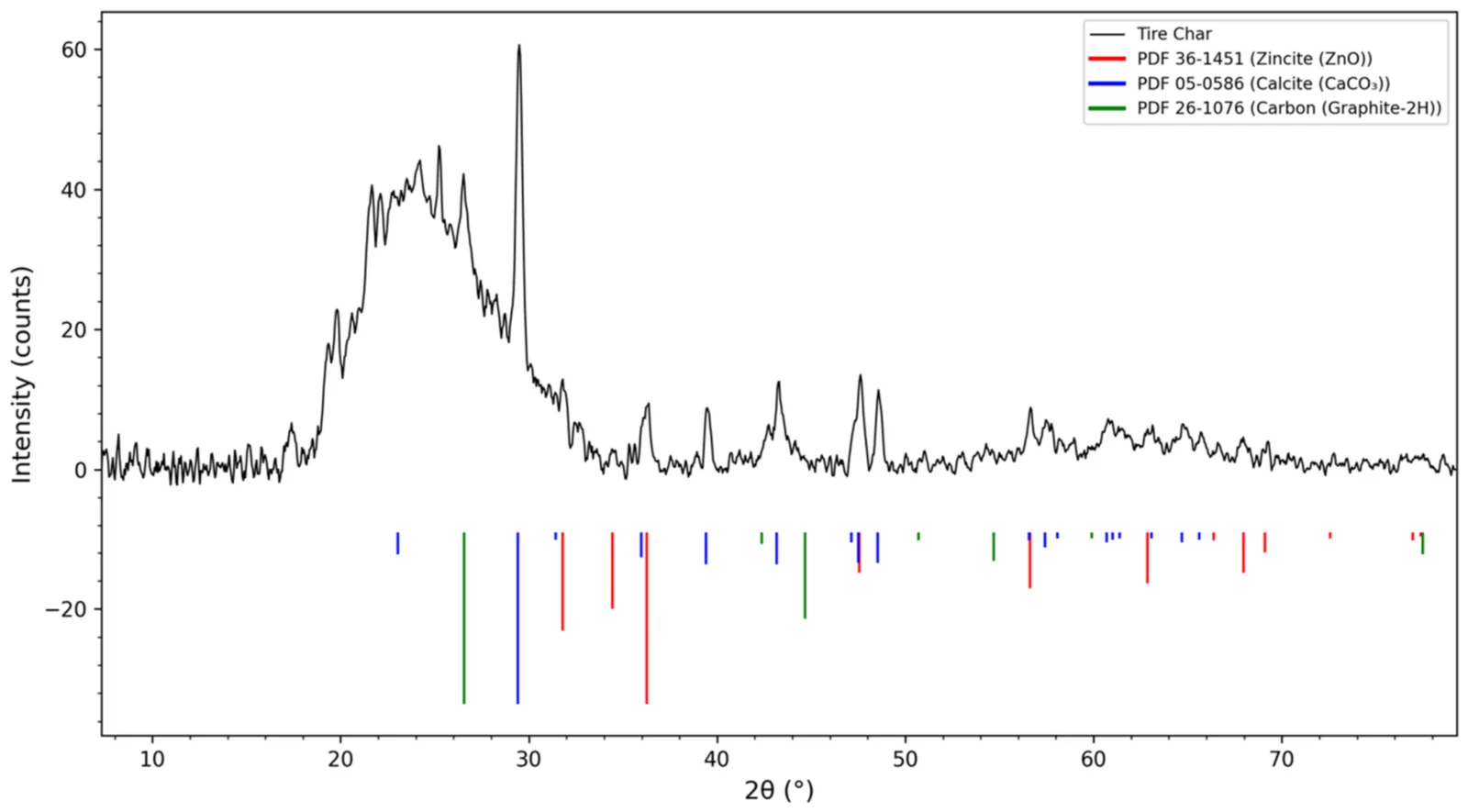
XRD of WTRC.

Figure 1 displays the XRD pattern of the WTRC. It shows a broad halo-centered peak between $2\theta =20^{\circ }$ and $27^{\circ }$, indicating the disordered, amorphous polymeric matrix characteristic of the vulcanized rubber matrix [18]. Composed of this amorphous halo are sharp, well-defined crystalline diffraction peaks corresponding to inorganic fillers. The adjective reflection appearing at $2\theta =29.5^{\circ }$ corresponds to the (104) plane of calcite (${\mathrm{CaCO}}_{3}$, JCPDS 96-901-6707), with secondary lines at $23.1^{\circ }$, $39.4^{\circ }$, and $48.5^{\circ }$ [19]. Additionally, crystalline peaks identified at $2\theta =31.8^{\circ }$, $34.4^{\circ }$, $36.3^{\circ }$, $47.5^{\circ }$, and $56.6^{\circ }$ are indexed to the zincite phase (ZnO, JCPDS 36-1451) [20]. These inorganic additives remain structurally intact within the ground rubber matrix despite mechanical processing, providing active polar sites that facilitate electrostatic and chemical interactions with ionic dye molecules.

#### 3.1.2. Thermal Stability and Degradation Behavior (Tga/Dtg & Dsc)

**Figure 2:**
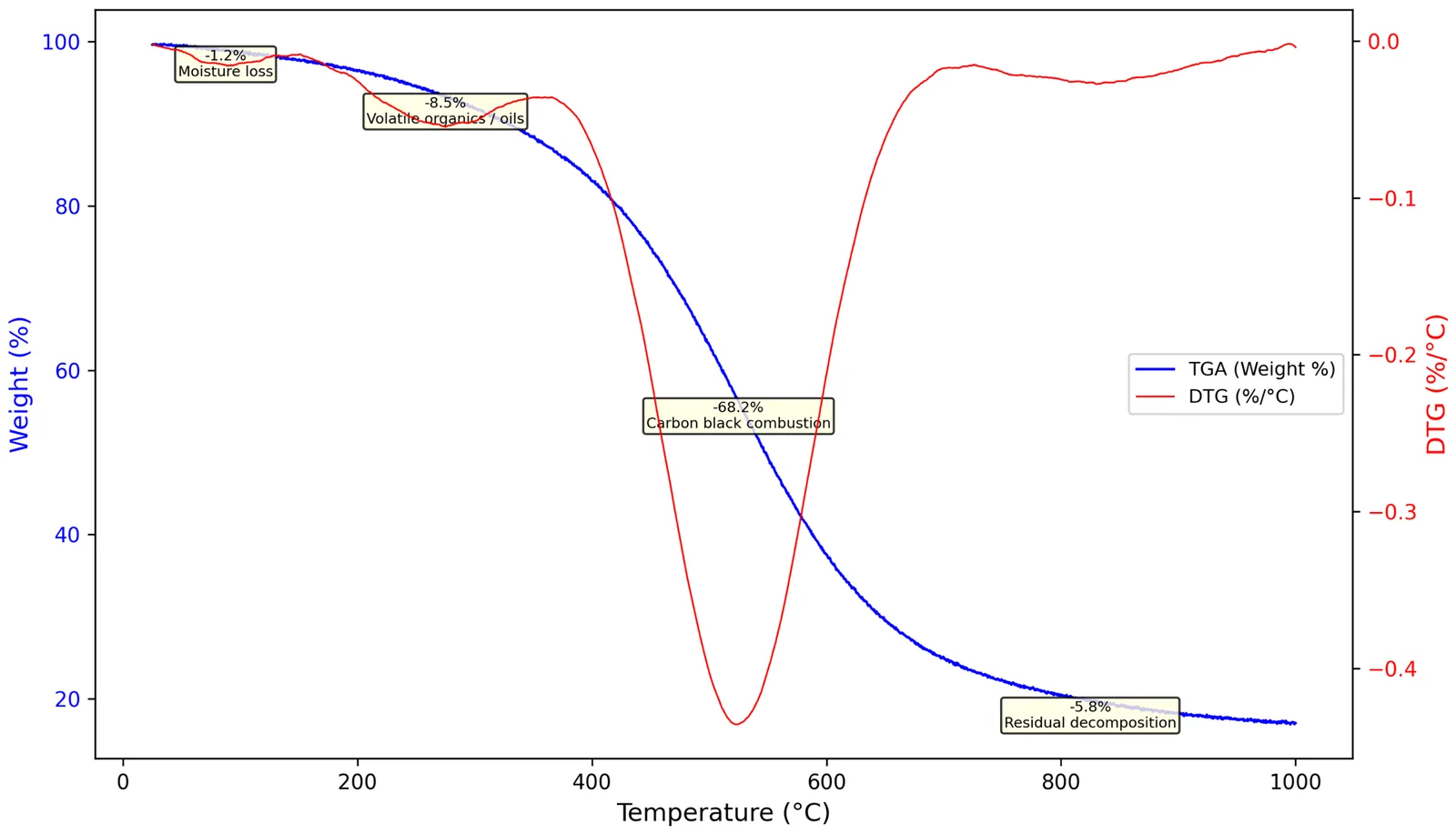
TGA/DTG of WTRC.

**Figure 3:**
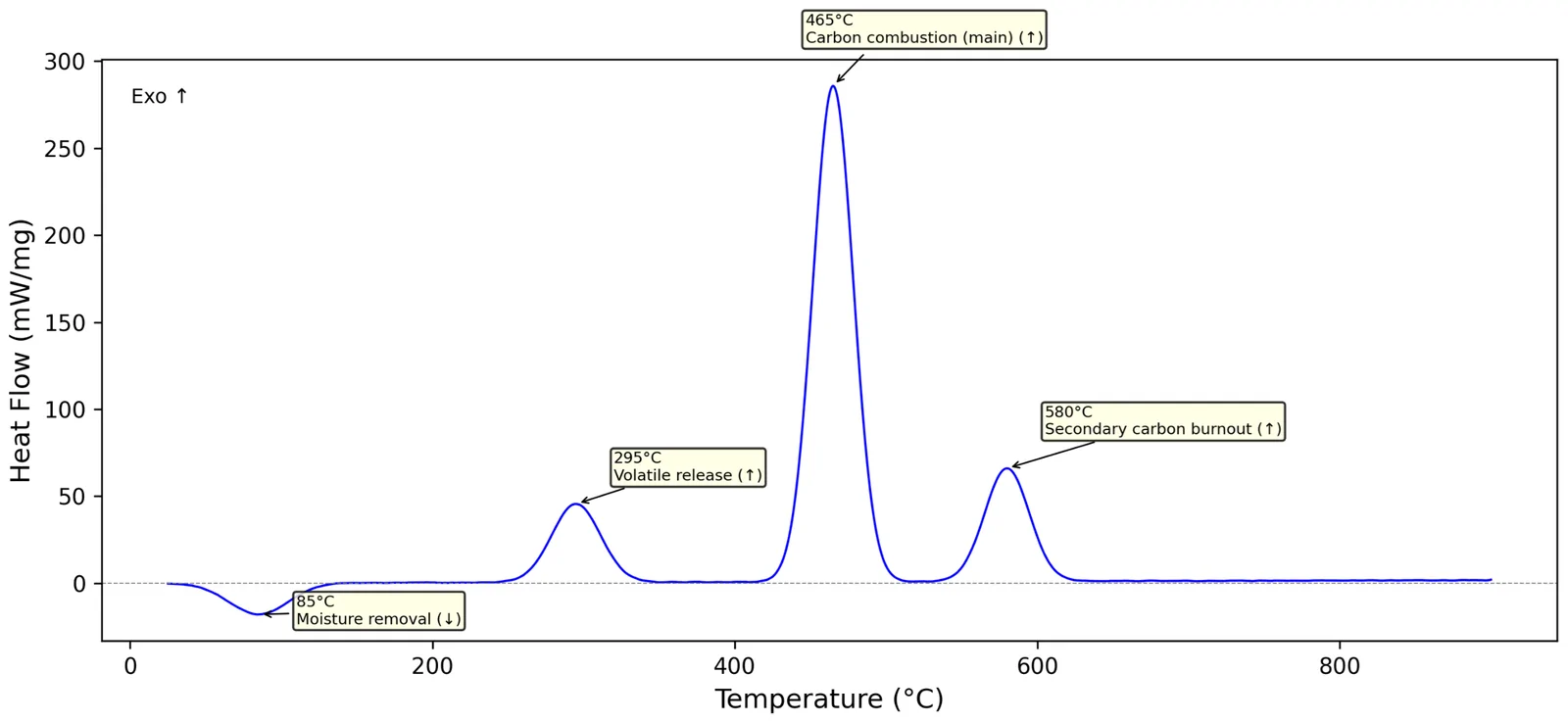
DSC of WTRC.

Thermal stability and degradation properties of the WTRC were assessed via thermogravimetric analysis/derivative thermogravimetry (TGA/DTG) under a nitrogen atmosphere (Figure 2) and differential scanning calorimetry (DSC) (Figure 3) up to 1000 °C. There are four distinctive weight losses and thermal transitions in the observed thermal behavior. At temperatures below 150 °C, there is a small weight loss of 1.2%, which is associated with the mild endothermic transition at 85 °C registered on the DSC diagram representing the physical moisture evaporation process [21]. Another weight loss of 8.5% takes place between 200 °C and 350 °C and corresponds to the exothermic transition at 295 °C, which is caused by the evaporation and thermal decomposition of organic processing oils and plasticizers [22]. Structural degradation is characterized by the highest weight loss of 68.2% in the range of 380–650 °C, which is registered as the maximal mass loss rate on the DTG graph near 525 °C coinciding with the exothermic combustion peak on the DSC plot at 465 °C, which is caused by the breakdown of the polymer matrix and carbon black burnout. Finally, a small weight loss of 5.8% at temperatures above 900 °C occurs alongside the second exothermic transition at 580 °C, which represents the decomposition of inorganic carbonate fillers, resulting in a final pyrolysis residue content of approximately 16.3% stable inorganic ash [23]. Hence, the WTRC demonstrates structural stability up to 200 °C and can be used for ambient water treatment.

#### 3.1.3. SEM and TEM Analysis

**Figure 4:**
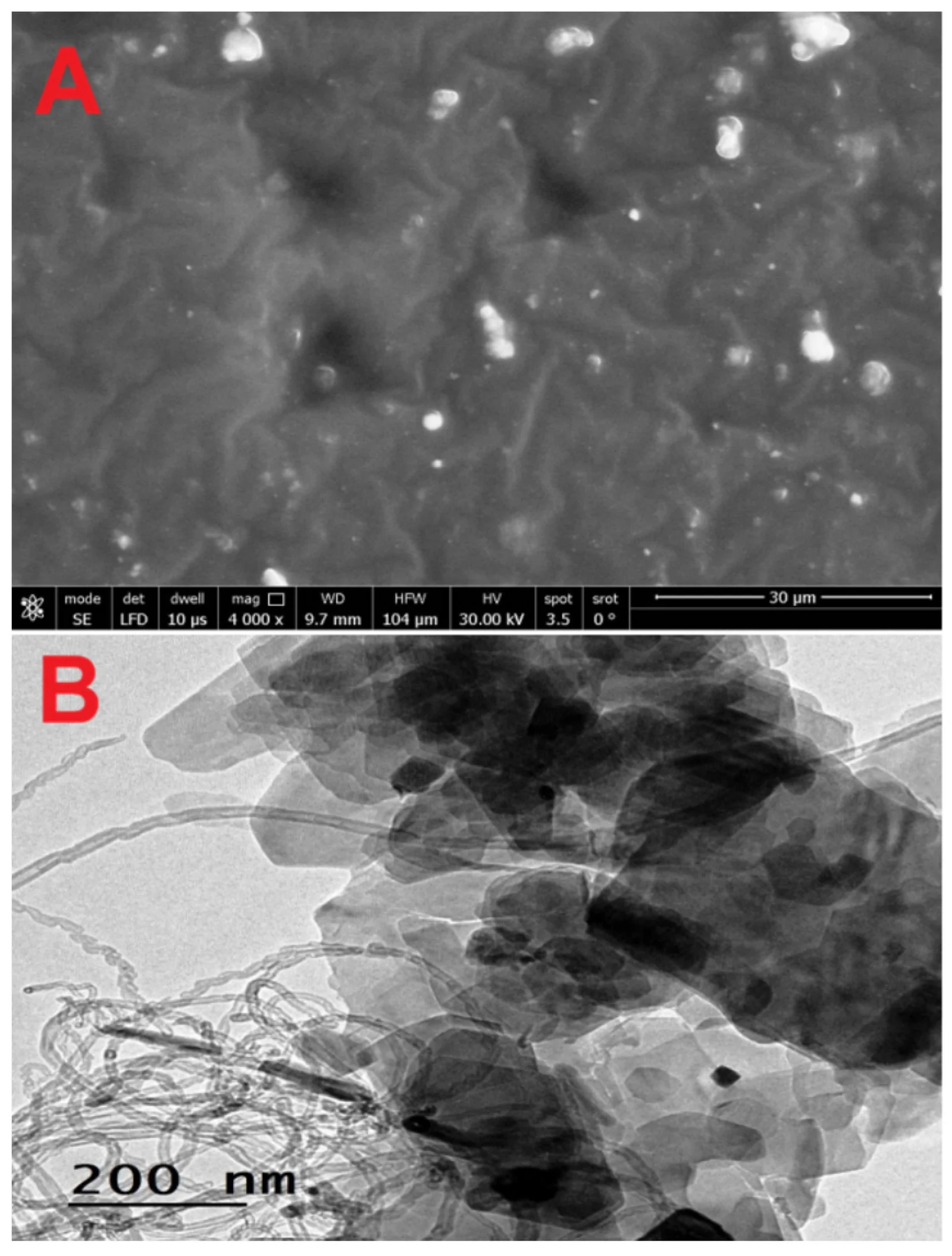
SEM (**A**) and TEM (**B**) images of WTRC.

The SEM micrograph of the WTRC, Figure 4A, depicts a rough and extremely irregular surface with micro-cavities and folds that make a large physical surface area. Bright sub-micron-sized metallic additives are evenly distributed on the dark polymeric matrix and create a heterogeneity at the interface [24]. At the nanometer scale, the TEM micrograph, Figure 4B at 200 nm, illustrates an intricate combination of thin overlapping carbon sheets and tubular filaments. Nano-sized aggregates are strongly intertwined in this cross-linked rubber matrix and are represented by carbon black clusters and inorganic nanoparticles. Overall, the presence of the micro-cavities in the SEM photo and high surface area sheets and tubes in TEM images makes the WTRC a robust structure for the surface interactions such as adsorption applications.

#### 3.1.4. Energy-Dispersive X-Ray Spectroscopy (EDX)

**Figure 5:**
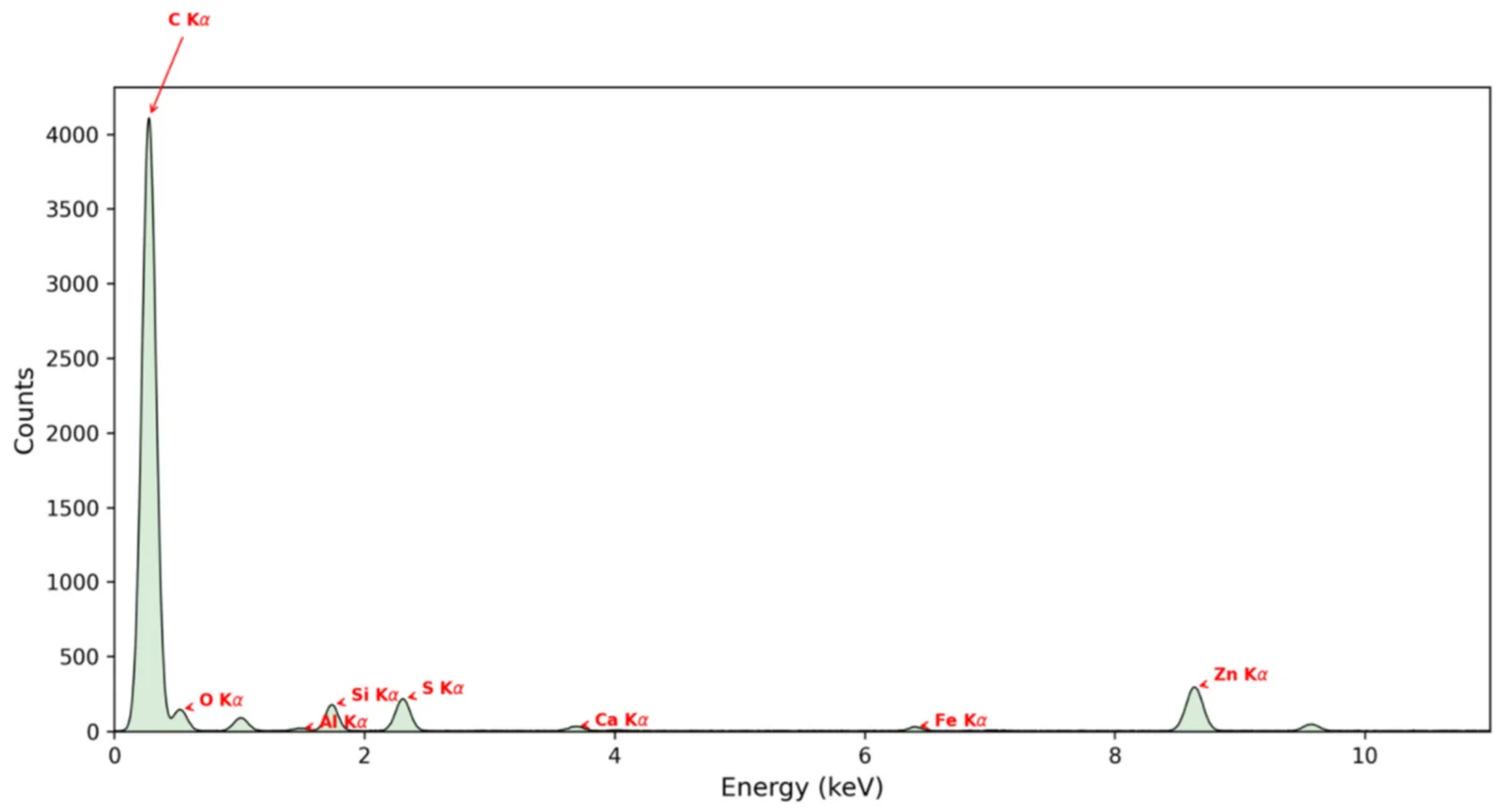
EDX spectrum of WTRC.

The EDX spectrum, Figure 5 shows that carbon (C) is the major element in the WTRC with the high intensity peak at 0.28 keV. The oxygen (O) and sulfur (S) elements peaks are observed, which account for the elements of the organic backbone as well as sulfur linkages due to the vulcanization process. Some minor peaks for inorganic elements like zinc (Zn), calcium (Ca), silicon (Si), aluminum (Al) and iron (Fe) can be observed that account for the inorganic compounds used in rubber tires production [25]. The elemental analysis of WTRC was performed using EDX, and the obtained results are listed in Table 1.

#### 3.1.5. Hydrodynamic Size Distribution and Zeta Potential Analysis

**Figure 6:**
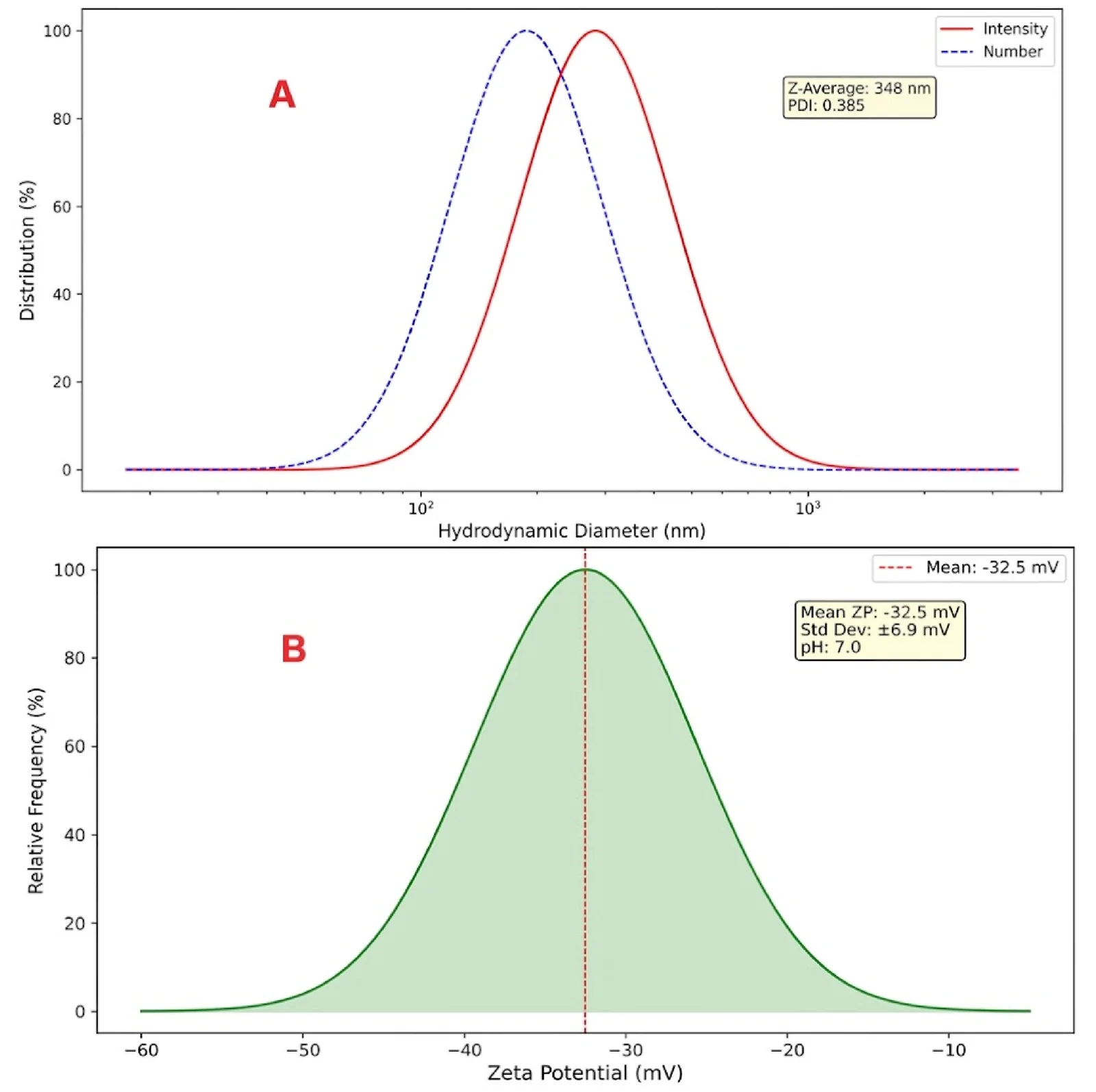
DLS (**A**) and Zeta potential (**B**) of WTRC.

Colloidal stability and surface charge characteristics of WTRC suspension were assessed by means of dynamic light scattering (DLS), Figure 6A and Zeta potential, Figure 6B analyses. As evident from the DLS plot, the particles of WTRC demonstrated a Z-Average hydrodynamic diameter of 348 nm with a Polydispersity Index (PDI) of 0.385, thus exhibiting a moderately wide sub-micron size distribution. The intensity-weighted plot (red solid line) was centered at 350 nm, due to a higher sensitivity of larger particle aggregates to the light-scattering effect. In contrast, the number-weighted plot (blue dashed line) had the maximum value around 200 nm, proving the dominance of smaller physical particles within sub-micron size range. This prevalence of fine particles is associated with an increased surface-to-volume ratio, which is highly advantageous for a fast liquid–solid mass exchange occurring during the dye’s adsorption process. To support the size data, the surface charge distribution of WTRC at pH 7.0 resulted in the presence of a remarkable mean Zeta potential of −32.5 mV (±6.9 mV). A value exceeding 30 mV proves the significant electrostatic repulsion between adjacent particles, which prevents their quick aggregation in aqueous suspension. Moreover, such pronounced negative surface charge can be attributed to the presence of oxygenated functional groups and inorganic compound fillers incorporated into the rubber matrix. Taking into account the application of this sorbent in the dye remediation process, the obtained negative surface potential creates a beneficial electrostatic field for cationic dye molecules. Therefore, the combination of DLS and Zeta potential parameters prove the structural and electrostatic stability of mechanically ground WTRC for MB adsorption.

### 3.2. Adsorption Studies

In order to assess the efficiency and effectiveness of the WTRC in wastewater treatment, batch adsorption tests have been performed by using MB as a pollutant. This section provides an overview of the detailed study of the operating parameters such as pH of solution, adsorbent dosage, initial dye concentration, and contact time. Additionally, the removal mechanism of the process has been explored using equilibrium isotherms, kinetics and surface studies.

#### 3.2.1. Effect of Adsorbent Dosage

**Figure 7:**
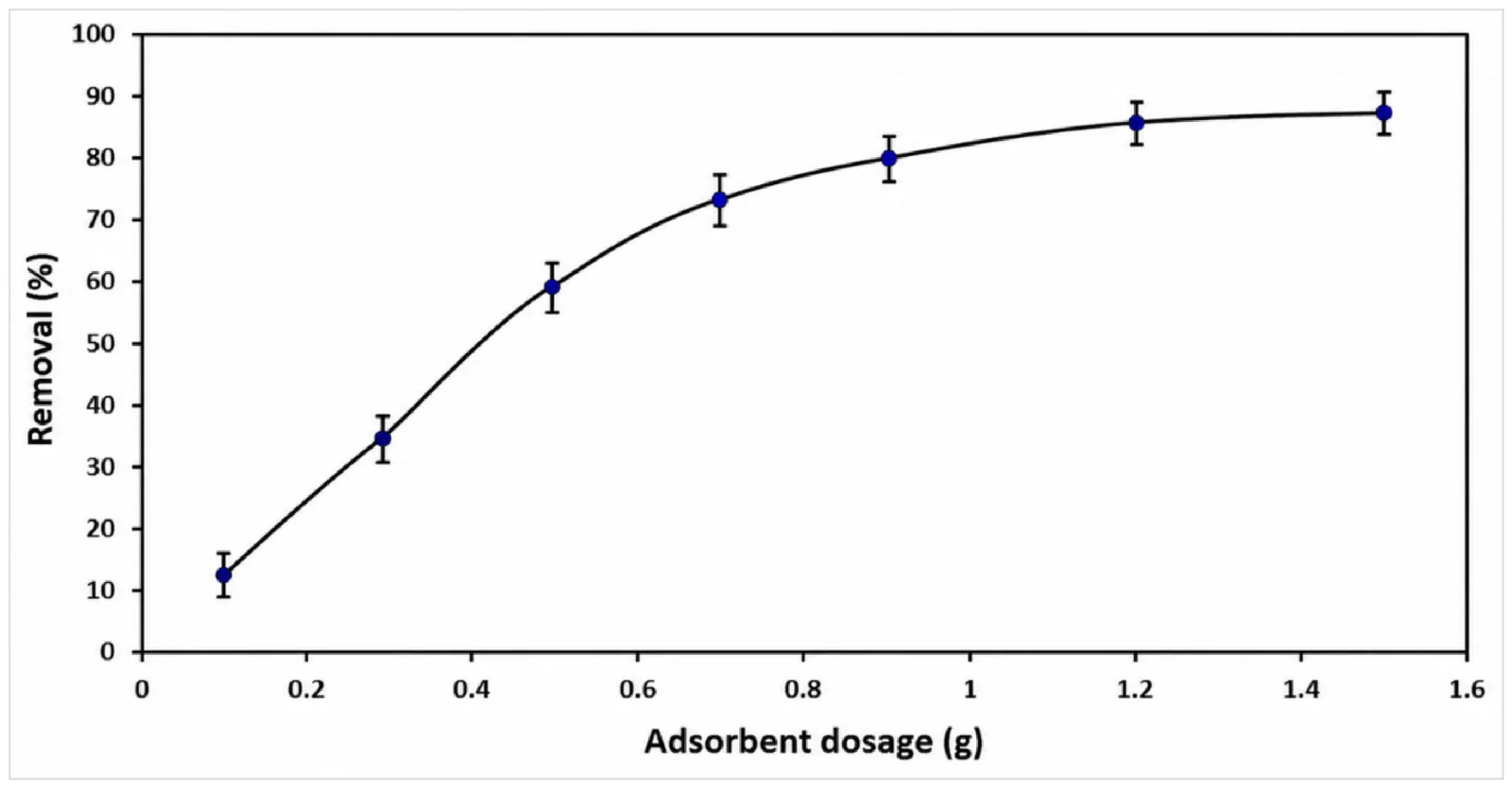
Effect of WTRC dosage on the removal percentage of MB (pH = 11, MB concentration = 5 mg/L, contact time = 30 min, and agitation speed = 300 rpm).

The effect of varying doses of the adsorbent, WTRC, in removing MB was determined over a range of 0.1 g to 1.5 g (in 50 mL of dye solution). As shown in Figure 7, an increase in the adsorbent dose from 0.1 g to 1.2 g resulted in a significant rise in the efficiency of removing MB, from ∼12% to ∼86%. The reason for such considerable enhancement is the increased surface area and the number of active binding sites on the WTRC. A further increase of the WTRC dose, from 1.2 g to 1.5 g, produced only an insignificant increase in the efficiency of the process up to ∼87.5% because of particle aggregation and the overlapping of the active sites, which cause a decrease in the effective specific surface area in relation to a constant volume of the adsorbent [26]. Thus, the most suitable adsorbent dose for further batch tests was found to be 1.2 g.

#### 3.2.2. Effect of Initial Solution pH

**Figure 8:**
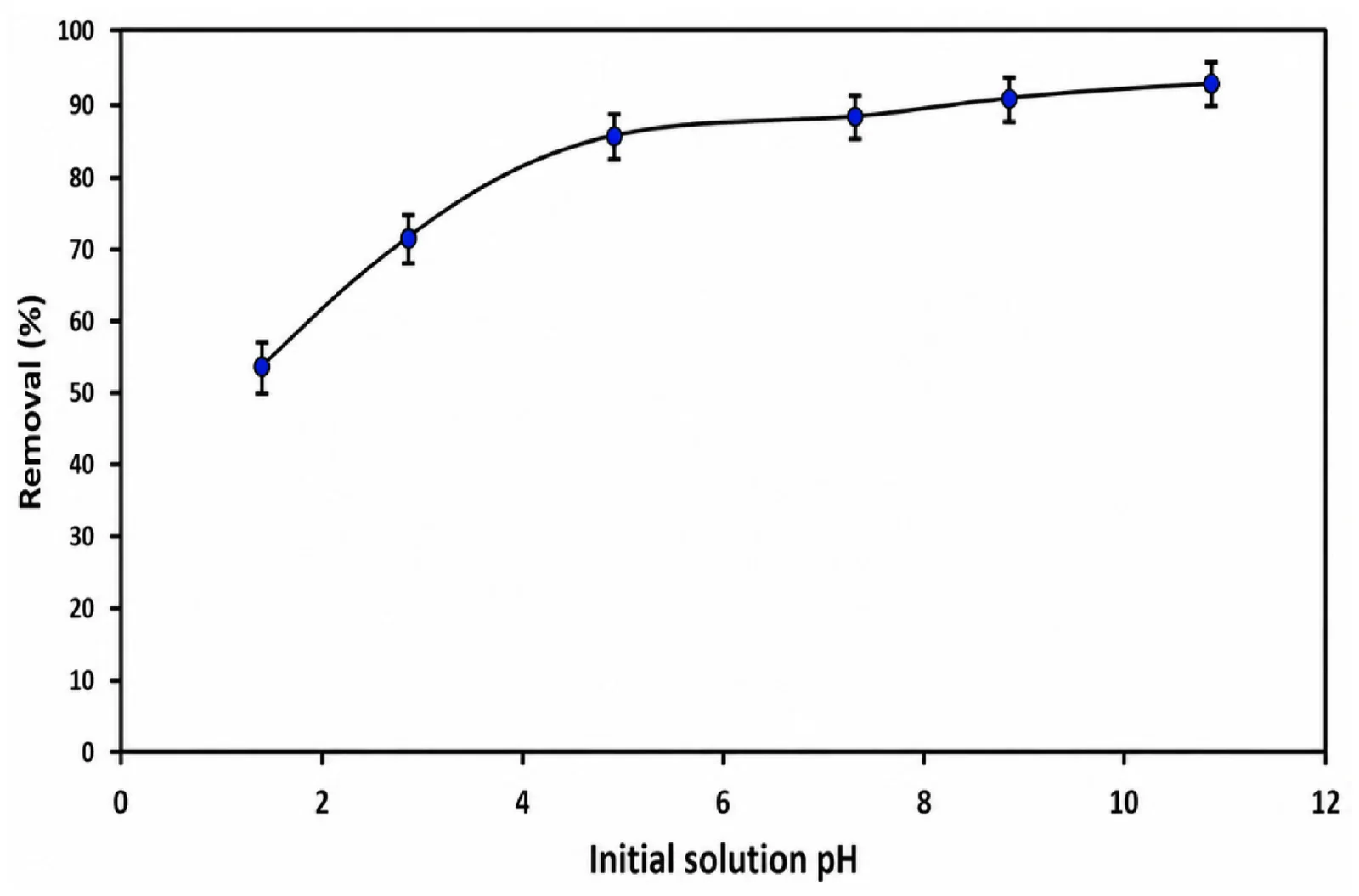
Effect of initial solution pH on the removal percentage of MB (MB concentration = 5 mg/L, contact time = 30 min, adsorbent dose = 1.2 g/50 mL, and agitation speed = 300 rpm).

The impact of solution pH at the beginning of adsorption of MB onto WTRC was studied in the pH range of 1.5 to 11.0. As indicated in Figure 8, the removal percentage of MB shows an evident positive dependence on pH, constantly growing from ~54.2% at pH 1.5 to the highest removal percentage of 92.7% at pH 11.0. This behavior is significantly regulated by the charge characteristics of WTRC and the ionic state of dye molecules in the aqueous medium. In highly acidic environments (for example, pH 1.5–3.0), owing to the high content of protons (H^+^), protonation of functional groups on the surface of WTRC takes place. Thus, a positive surface charge state is created, and fierce competition between H^+^ and MB dye molecules for binding sites is developed. This leads to electrostatic repulsion and less efficiency of adsorption. With the increase of pH to neutral and basic environments (pH 5.0–11.0), progressive deprotonation of oxygen-containing functional groups in the WTRC matrix takes place [27]. Thus, the charge state becomes more negative and corresponds to the strong negative zeta potential for WTRC (−32.5 mV at pH 7.0). This gives the opportunity for strong electrostatic interaction with cationic MB molecules, hence the high removal efficiency at pH 11.0.

#### 3.2.3. Effect of Initial Dye Concentration

**Figure 9:**
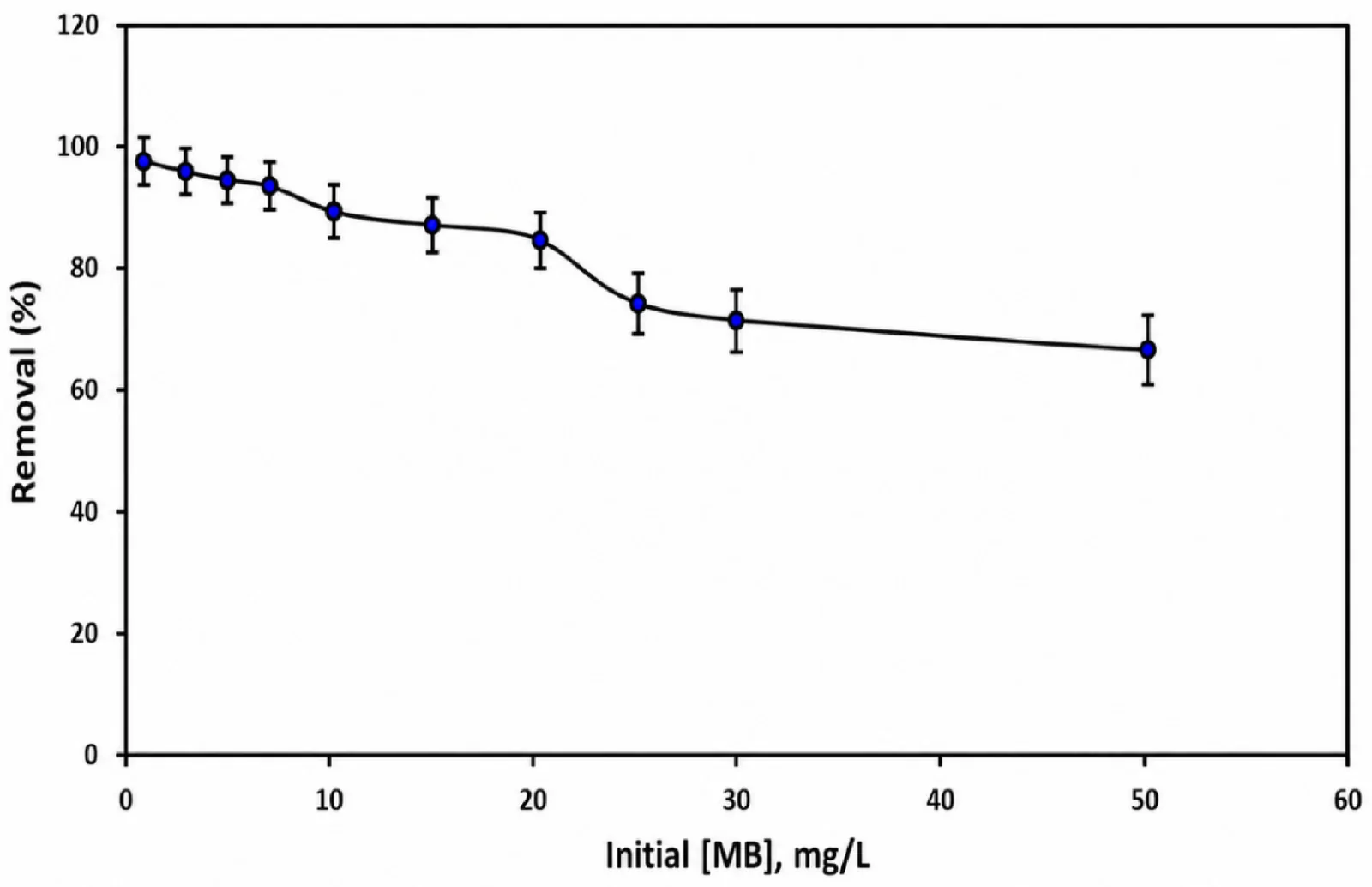
Effect of initial concentration of MB on the removal percentage of MB (contact time = 30 min, adsorbent dose = 1.2 g/50 mL, pH = 11, and agitation speed 300 = rpm).

The influence of initial dye concentration on MB removal efficiency by WTRC in comparison to the total number of dye molecules was studied over the concentration range of 1 mg/L to 50 mg/L. As shown in Figure 9, the removal percentage of MB shows a gradual decrease with an increase in initial dye concentration from ∼96.2% at 1 mg/L to ∼65.6% at 50 mg/L. Such a decrease in removal percentage with the increase in initial dye concentration is characteristic of batch adsorption. At lower initial concentrations of MB dye (1–5 mg/L), the number of active adsorption sites of WTRC, in comparison to the total number of dye molecules, is very high. Thus, almost all dye molecules obtain access to empty active sites, resulting in a maximum removal percentage. At higher initial MB dye concentrations (10–50 mg/L), with the same dose of WTRC, there is a limited number of surface-active sites, which gradually become saturated. The rest of the MB molecules remain unabsorbed due to steric hindrance and site exhaustion. This results in reduced removal percentage [28]. However, higher initial dye concentrations provide a greater concentration gradient, which helps overcome mass transfer resistance between the two phases.

#### 3.2.4. Effect of Contact Time

**Figure 10:**
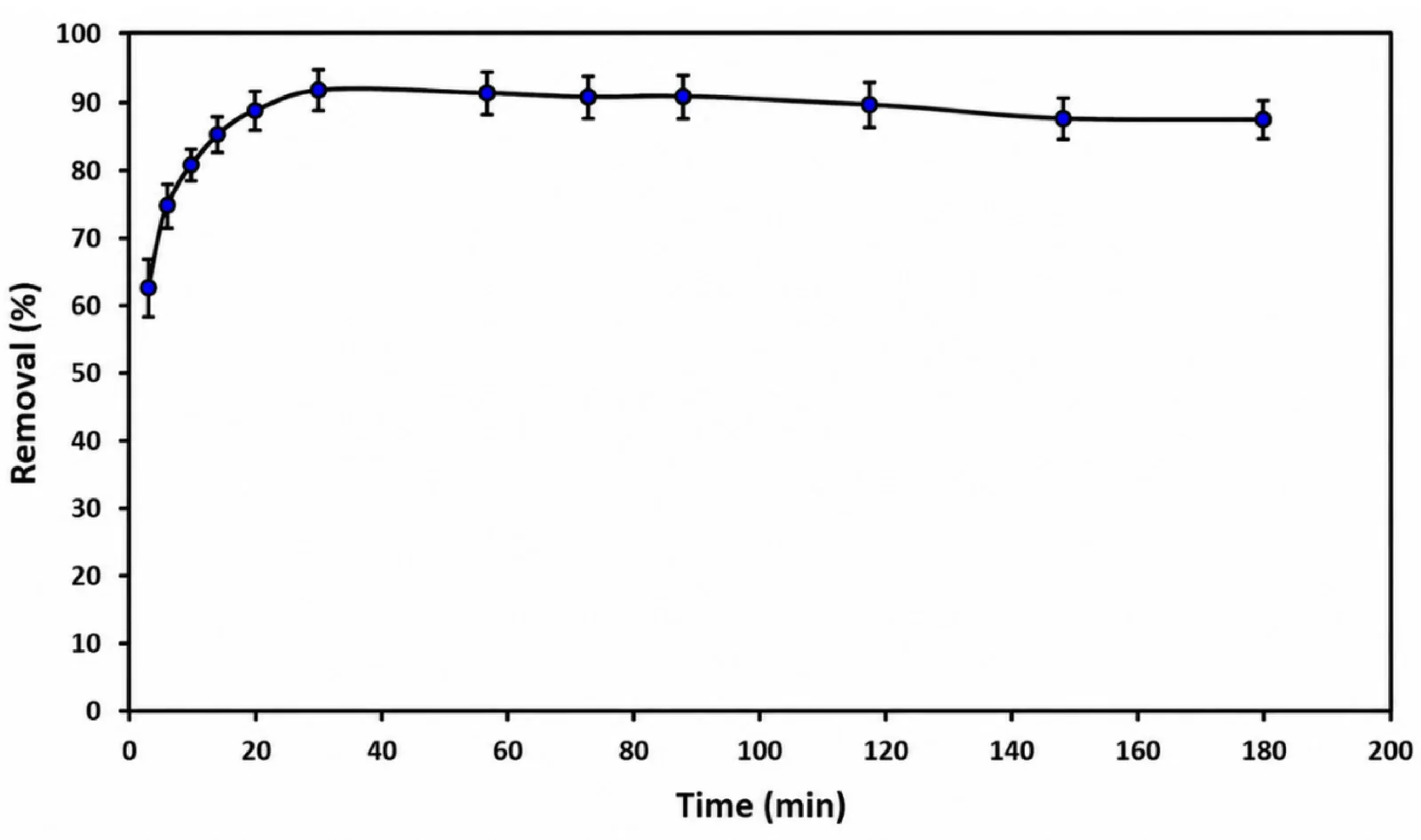
Effect of contact time on the removal percentage of MB (pH = 11, MB concentration = 5 mg/L, adsorbent dose = 1.2 g/50 mL, and agitation speed 300 rpm).

The influence of contact time on the MB removal efficiency by the WTRC was studied during a long period ranging from 3 to 180 min. Figure 10 depicts the rapid removal of MB during the initial stages and the gradual attainment of equilibrium. During the first 5 min of adsorption, MB removal quickly approached ∼76.8%, continuously moving toward a maximum of 92.2% at 30 min. When the contact time was more than 30 min and up to 180 min, there was no further change in the rate of removal, and the plateau region (∼88.0–91.5%) showed that the adsorption was in equilibrium. This rapid initial phase is due to the large number of unoccupied and easily accessible active binding sites and the steep concentration gradient leading to dye molecules being pulled towards the adsorbent. As the contact time increased, the vacant active sites were progressively filled, leading to steric hindrance and electrostatic repulsion between already adsorbed MB molecules and the incoming dye molecules [29]. Thus, 30 min was found to be the optimum contact time for attaining equilibrium.

#### 3.2.5. Isothermal Studies

**Figure 11:**
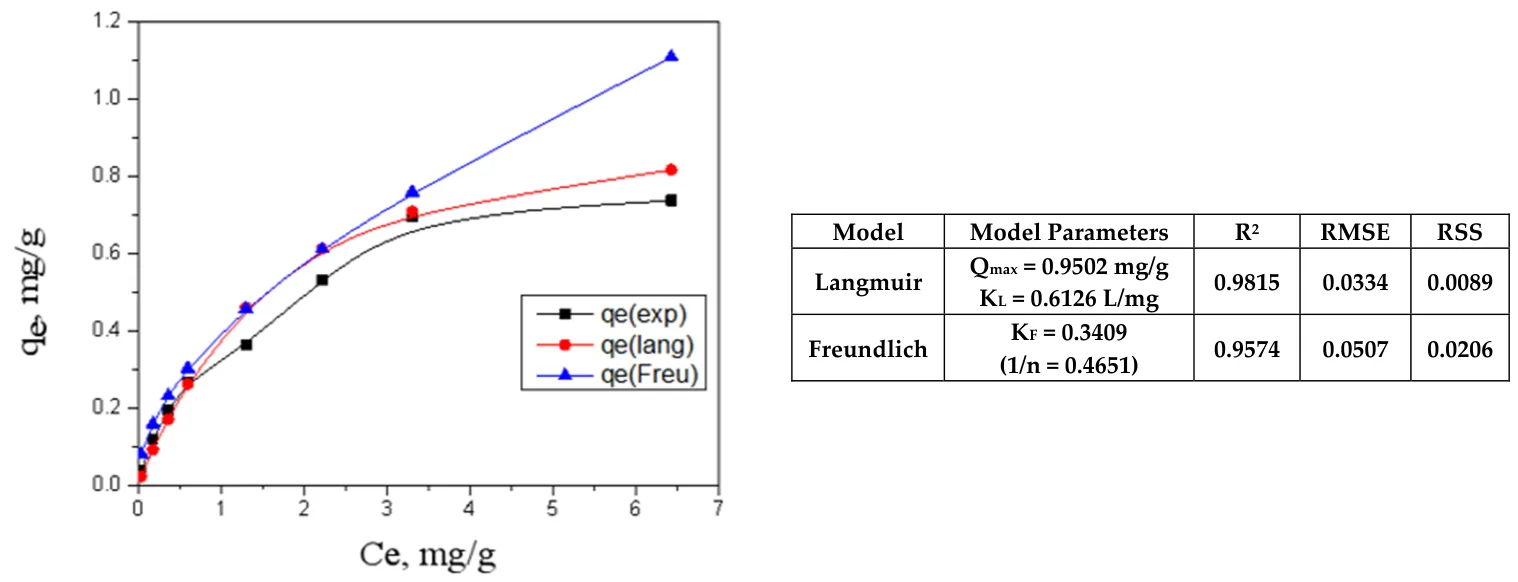
Non-Linearized Freundlich and Langmuir isotherm plots for MB adsorption onto WTRC.

Experimental equilibrium data on MB adsorption on WTRC were analyzed by fitting the nonlinear models Freundlich and Langmuir [30,31,32] (Figure 11); the Langmuir model gave the best fit with R^2^ = 0.982, which was more than Freundlich model, R^2^ = 0.957. The good fit of the Langmuir model indicates that adsorption predominantly occurred as a monolayer on relatively homogeneous adsorption sites on the surface of WTRC.

The theoretical monolayer capacity (q_max_) is calculated as 0.95 mg/g from Langmuir isotherm.

#### 3.2.6. Kinetic Studies

**Figure 12:**
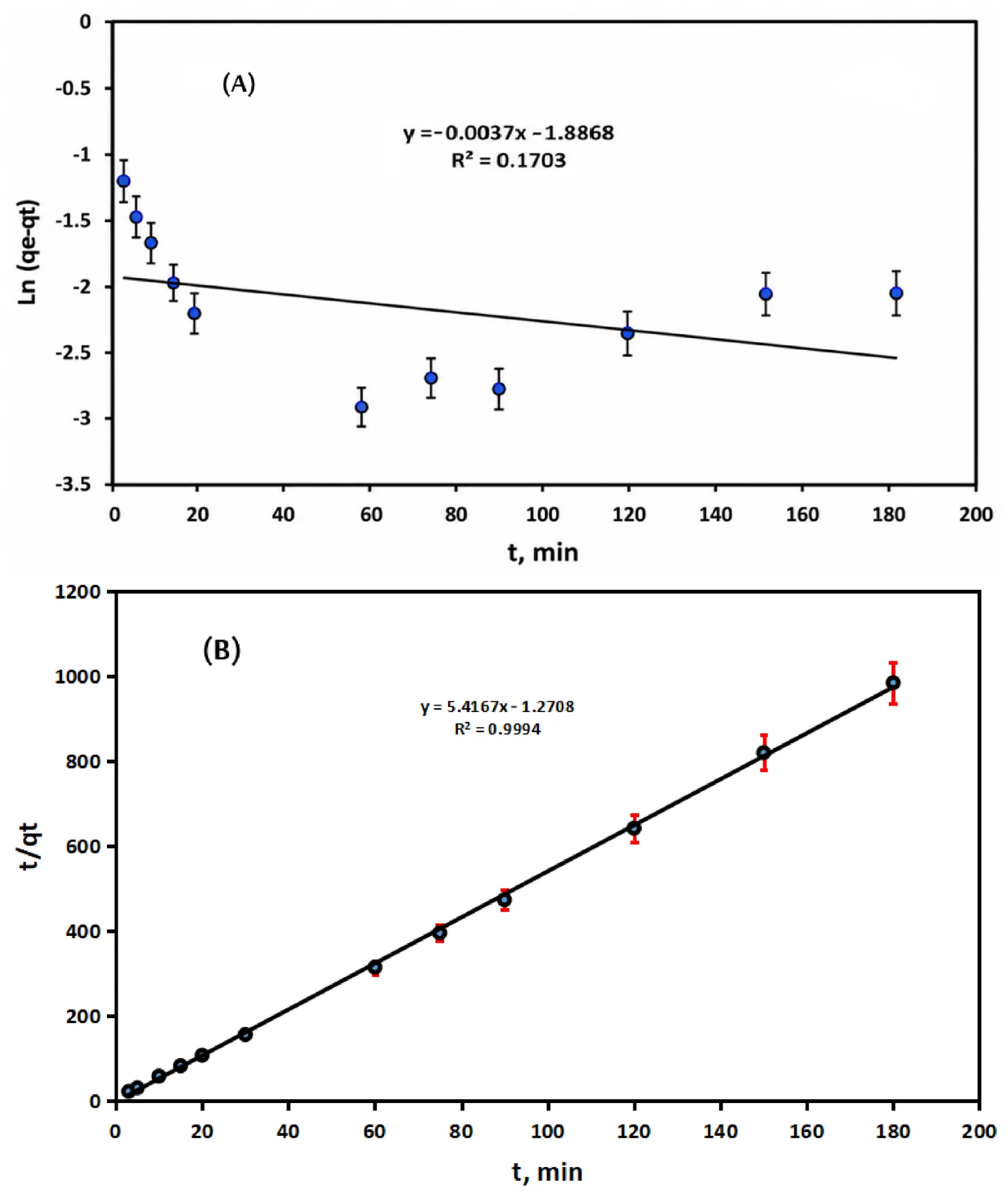
Pseudo-first-order (**A**) and pseudo-second-order (**B**) kinetic models for (MB) adsorption onto WTRC.

In order to evaluate the rate-controlling mechanism for MB adsorption onto WTRC, the kinetic data were plotted against PFO [33] and PSO [34,35] models (Figure 12A and Figure 12B, respectively). The PFO model exhibited a very weak linear fit (R^2^ = 0.1703), suggesting that physisorption is not involved in the rate-controlling step. In contrast, the PSO model gave almost a perfect linear fit (R^2^ = 0.999). Calculated equilibrium capacity ($q_{e,\mathrm{cal}}=0.185\;\mathrm{mg}/g$) from the slope of the line agrees well with the experimental results.

#### 3.2.7. Surface Morphology and Chemical Interaction Analysis After Adsorption

**Figure 13:**
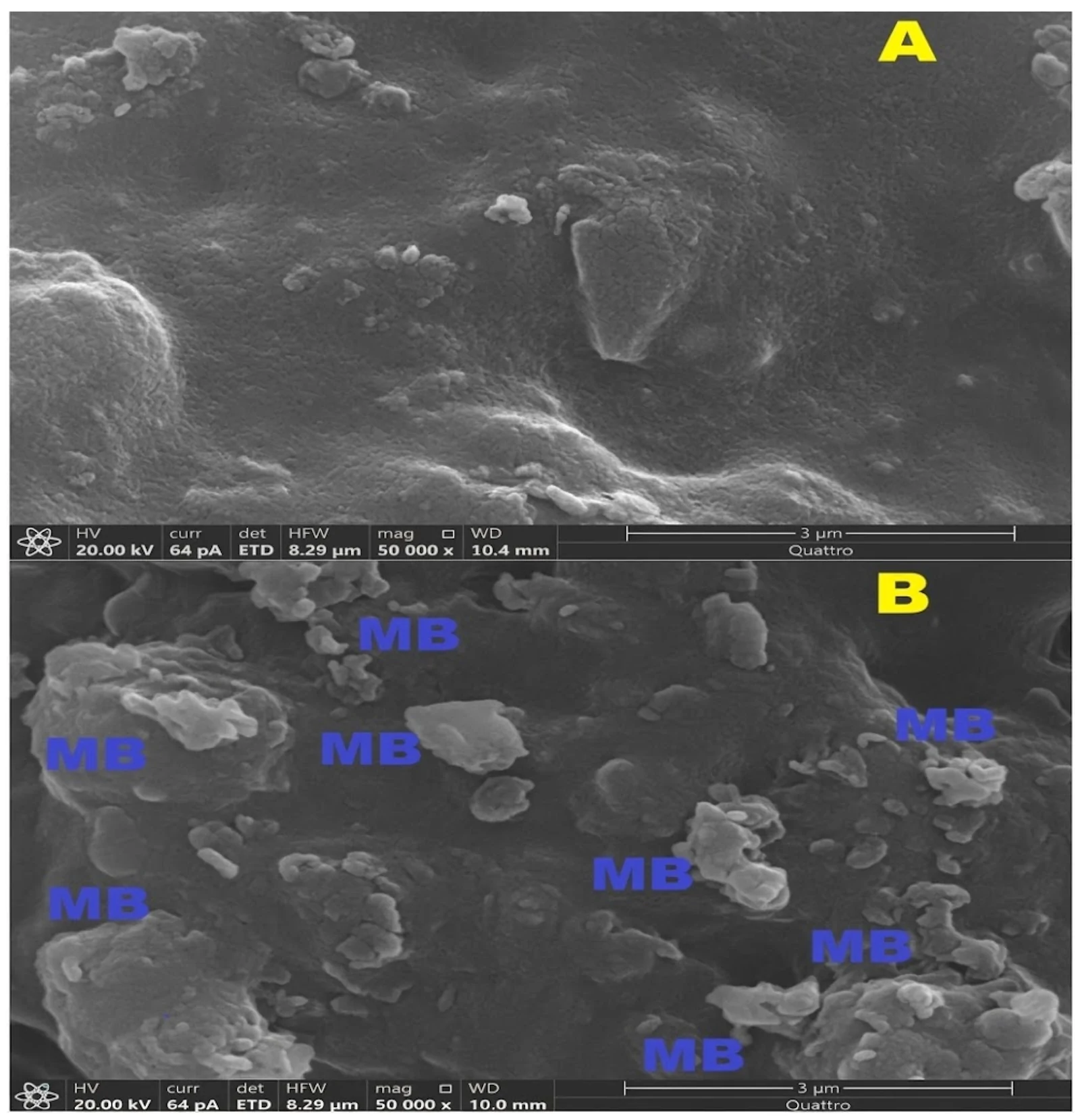
SEM images of WTRC before (**A**) and after (**B**) adsorption.

**Figure 14:**
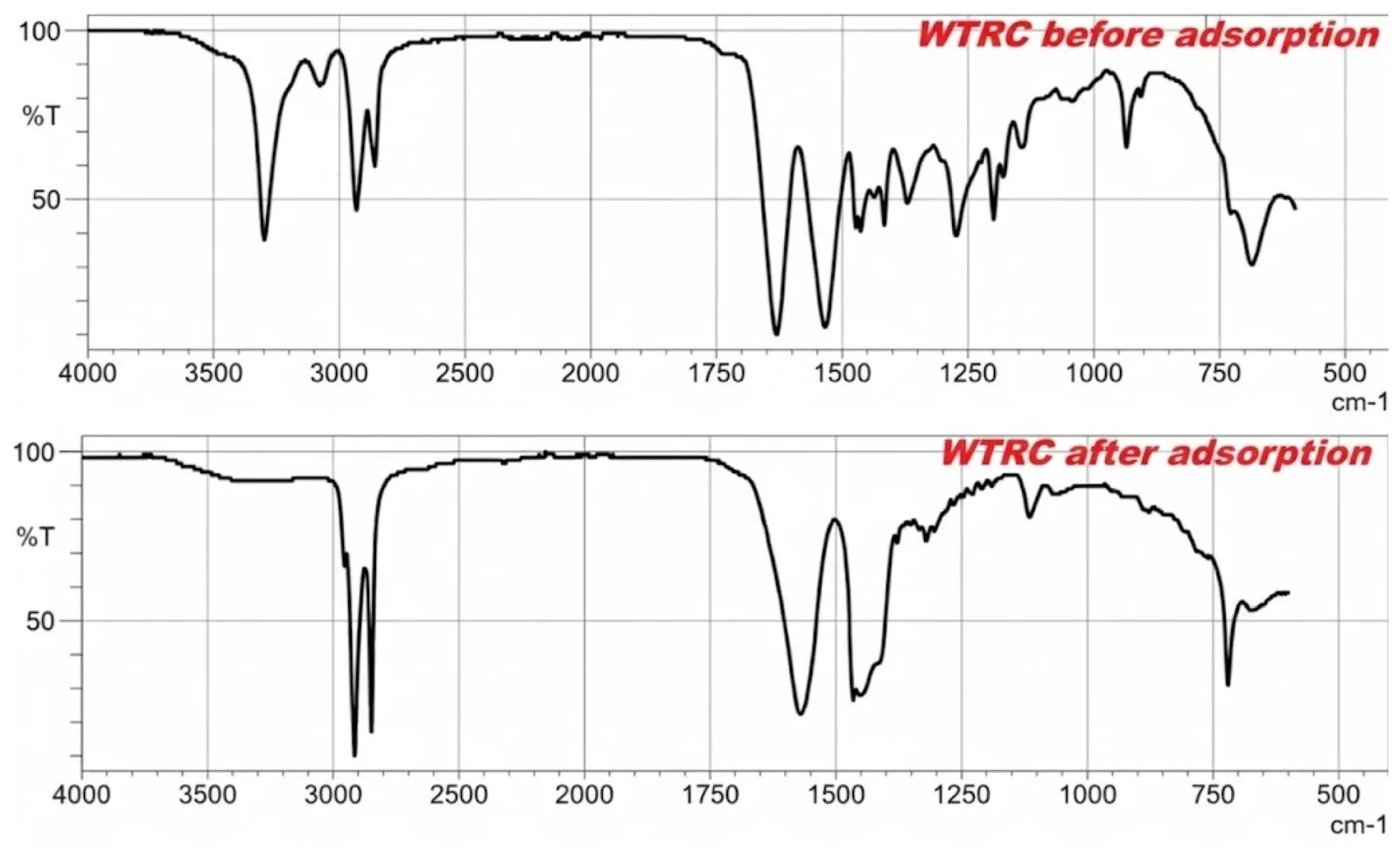
FTIR images of WTRC before and after MB adsorption.

In order to understand the interactions of MB with WTRC, the SEM post-adsorption microstructural examination, Figure 13, and the FTIR spectroscopic investigations, Figure 14, were carried out. Before the adsorption process, the SEM picture of WTRC shows a relatively continuous and rough surface of the polymeric material with visible micro-cavities. After the adsorption process, the SEM images show clear changes in structure, and agglomeration of MB molecular clusters, which were successfully deposited directly on the surface of the matrix. The FTIR spectra of the virgin WTRC material show typical absorption bands, among which the most prominent are the broad band of O-H/N-H stretching around 3300 cm^−1^; the C-H aliphatic stretching vibration (2850–2920 cm^−1^); and the fingerprint region, which is related to the rubber vulcanization network. After the MB loading, the intensity of the peak at 3300 cm^−1^ has significantly decreased, along with the appearance of some changes in intensity and position of the peaks in the 1000–1600 cm^−1^ region. Table 2 presents the analysis of the significant IR bands of WTRC and the changes in these bands after MB adsorption.

#### 3.2.8. Adsorption Mechanism

**Figure 15:**
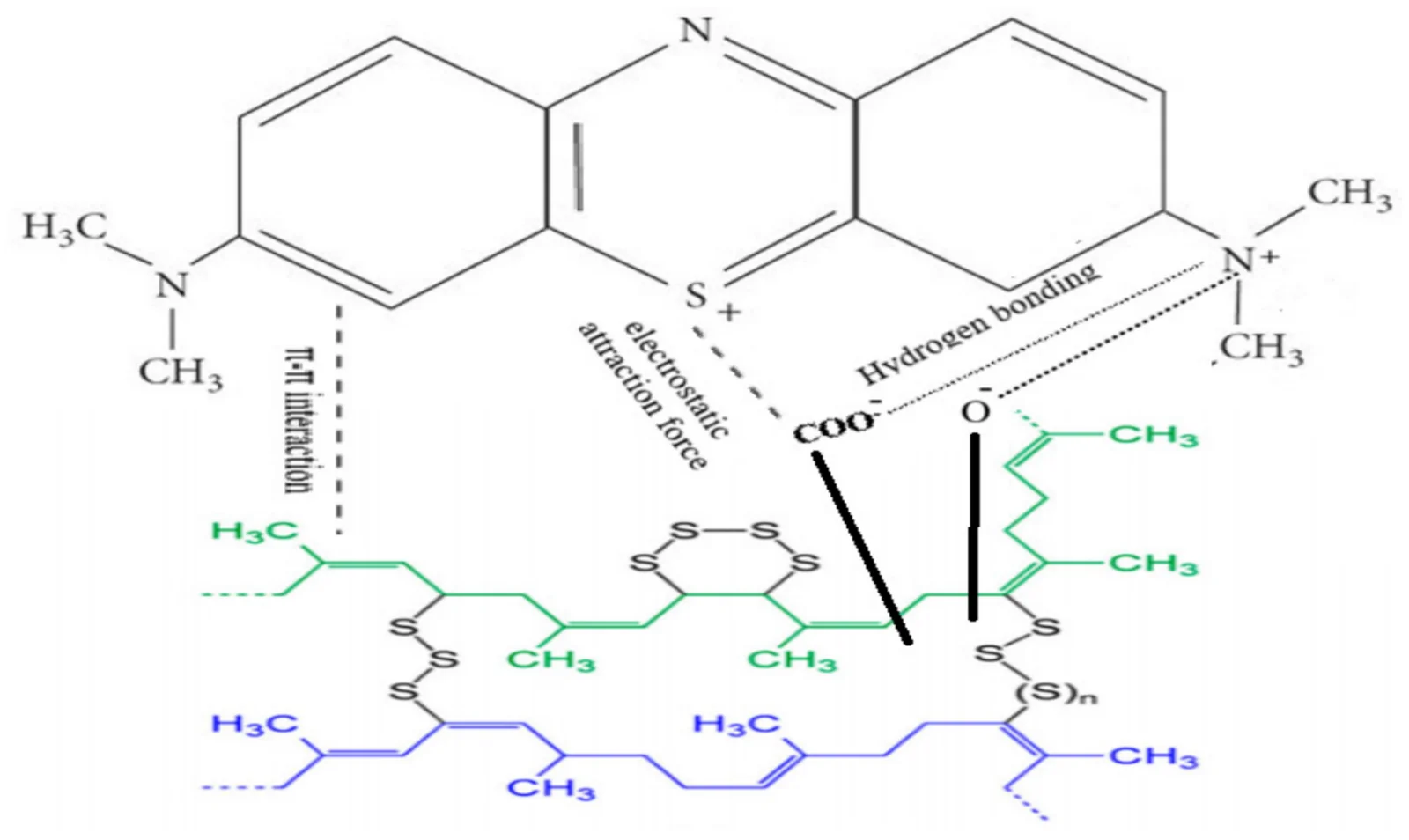
The possible interactions between MB dye and WTRC.

The mechanism of adsorption [36,37] of MB on the WTRC, Figure 15 may be interpreted according to the following pathways: (i) Electrostatic interactions of MB on the WTRC surface, MB as a cationic dye, and a negatively charged WTRC result from the negative functional groups (carboxylate, hydroxyl, and oxidized sulfur species). (ii) π-π interactions of the conjugated aromatic ring systems in MB and WTRC. (iii) Hydrogen bonding that may arise between MB and the rubber surface. (iv) The porous structure of WTRC may provide cavities that enable MB molecules to diffuse into these pores.

#### 3.2.9. Comparative Studies

The adsorption capacity of WTRC for MB removal was evaluated through comparison with a range of adsorbents reported in previous studies [38,39,40,41,42,43,44], including fly ash, glass, and activated carbon produced from agriculture and industrial waste recycling, Table 3.

## 4. Conclusions

This work illustrates the potential use of pure recycled tire crumb as an environmentally friendly adsorbent to remove MB dye from wastewater. Material characterization by XRD, EDX, TGA/DSC, SEM, TEM, DLS, and zeta potential analysis indicated a stable carbon-based matrix consisting of inorganic components with a strong negative surface charge (−32.5 mV at pH 7.0) and high colloidal stability. The adsorption tests showed the maximum efficiency (∼92.7%) of dye removal at pH (11.0), dose (1.2 g/50 mL), initial concentration (5 mg/L) of MB dye, and 30 min contact time. Analysis of equilibrium data revealed agreement with the Langmuir isotherm model (R^2^ = 0.982) and indicated monolayer adsorption on homogeneous adsorption sites. Adsorption kinetics followed the pseudo-second-order equation (R^2^ = 0.999). All the possible interactions between MB and WTRC were proposed. Therefore, the use of WTRC may be considered a sustainable route to treat industrial dye with less energy consumption for pyrolysis or chemical activation.

## Figures and Tables

**Table 1:** Quantitative elemental composition of WTRC.

Element	Weight %	Atomic %
Carbon (C)	72.45	84.49
Oxygen (O)	11.35	9.94
Zinc (Zn)	6.85	1.46
Silicon (Si)	3.42	1.71
Sulfur (S)	2.88	1.26
Calcium (Ca)	1.46	0.51
Aluminum (Al)	0.86	0.45
Iron (Fe)	0.73	0.18

**Table 2:** Major FTIR absorption bands of WTRC before and after MB adsorption.

Functional Group/Assignment	Wavenumber Before (cm^−1^)	Wavenumber After (cm^−1^)	Spectral Change and Mechanism
–OH/–NH Stretching	~3300	Flattened/Broad	Disappearance/flattening due to H-bonding
Aromatic C-H Stretching	~3030	Flattened/Merged	Disappearance attributed to aromatic π–π stacking
Aliphatic -${\mathrm{CH}}_{2}$ Stretching	2920, 2851	2918, 2849	Sharp intensification in peak depth
Aromatic C=C Stretching	1632	1588	Significant shift ($\Delta \nu =44{\;\mathrm{cm}}^{-1}$) indicating chemical binding
Fingerprint Bands($C\text{–}O,\;C\text{–}S,\;C\text{–}N,\text{–}{\mathrm{CH}}_{2}$ deformation)	1450–1000	Flattened/Disappeared	Disappearance of multiple fine peaks due to MB surface masking layer
Aromatic C-H Bending (Out-of-plane)	690	715	Peak shift in the fingerprint region

**Table 3:** Comparison of the maximum adsorption capacities (Q_max_) of WTRC and other adsorbents for MB dye adsorption.

Adsorbent	Q_max_, mg/g	Ref.
Woody fly ash	0.67	[38]
Biomass fly ash	0.62	[39]
Coal fly ash	0.07–0.12	[40]
Corncob based activated carbon	0.8	[41]
Glass wool	2.24	[42]
Almond shell-activated carbon	1.3	[43]
Activated carbon from Tamarind seeds	1.42	[44]
WTRC	0.95	Current work

## Data Availability

The original contributions presented in this study are included in the article. Further inquiries can be directed to the corresponding authors.
